# Research progress on antiviral constituents in traditional Chinese medicines and their mechanisms of action

**DOI:** 10.1080/13880209.2022.2074053

**Published:** 2022-05-28

**Authors:** Zhi Chen, Si-yong Ye

**Affiliations:** a Pharmaceutical College, Shandong University of TCM, Jinan, People’s Republic of China; b Department of Pharmacy, Jinan Second People's Hospital, Jinan, People’s Republic of China

**Keywords:** Active ingredients, antiviral, mechanism of action, anti-inflammatory effect, traditional Chinese medicines

## Abstract

**Context:**

Viruses have the characteristics of rapid transmission and high mortality. At present, western medicines still lack an ideal antiviral. As natural products, many traditional Chinese medicines (TCM) have certain inhibitory effects on viruses, which has become the hotspot of medical research in recent years.

**Objective:**

The antiviral active ingredients and mechanisms of TCM against viral diseases was studied in combination with the pathogenesis of viral diseases and antiviral effects.

**Materials and methods:**

English and Chinese literature from 1999 to 2021 was collected from databases including Web of Science, PubMed, Elsevier, Chinese Pharmacopoeia 2020 (CP), and CNKI (Chinese). Traditional Chinese medicines (TCM), active ingredients, antiviral, mechanism of action, and anti-inflammatory effect were used as the key words.

**Results:**

The antiviral activity of TCM is clarified to put forward a strategy for discovering active compounds against viruses, and provide reference for screening antivirus drugs from TCM. TCM can not only directly kill viruses and inhibit the proliferation of viruses in cells, but also prevent viruses from infecting cells and causing cytophilia. It can also regulate the human immune system, enhance human immunity, and play an indirect antiviral role.

**Discussion and conclusion:**

Based on the experimental study and antiviral mechanism of TCM, this paper can provide analytical evidence that supports the effectiveness of TCM in treating virus infections, as well as their mechanisms against viruses. It could be helpful to provide reference for the research and development of innovative TCMs with multiple components, multiple targets and low toxicity.

## Introduction

Viruses are a class of highly infectious intracellular parasitic microorganisms, which seriously endanger human and animal health. Western antiviral medicines, including nucleoside analogues, reverse transcriptase inhibitors, neuraminidase inhibitors, etc., exert their antiviral functions mainly by inhibiting viral processes such as adsorption, invasion, nucleic acid synthesis, and uncoating (Shahabadi and Moshtkoob 2020; Kaczmarek et al. 2021; Taieb et al. 2021). The problems with these antiviral agents, however, are the development of drug resistance due to viral mutations, the functional inability caused by viral incubation, the evident adverse reactions, as well as the complex therapeutic regimes (Amarelle et al. 2017). TCM has been used effectively to combat epidemics infection, and saved many lives. So far, hundreds of TCM prescriptions have been developed for the prevention and treatment of infectious diseases and it is now credited for the successful battle against COVID-19 in China (Zheng, Baak, et al. 2020; Lee et al. 2021). TCM with their unique perspectives, emphasize the interactions with viruses and organisms, which have comprehensive effects such as inhibiting viral replication, preventing virus-induced cytopathy, and regulating immunity (Chen, Zhong, et al. 2019; Shen et al. 2019; Huang et al. 2020). With the continuous emergence of large-scale screening technologies for antiviral agents, the development of highly effective antiviral TCM has become a current hot topic in drug R&D.

In TCM, infectious diseases caused by viruses are referred to as ‘plagues’. The pathogenic mechanisms of viruses mainly include the direct damage or apoptosis of host cells, the alteration of normal cellular functions, the induction of excessive inflammation or pathological injury, the immunosuppression, etc. Adhering to the syndrome differentiation-based treatment in TCM, these viral infectious diseases are often treated with heat-clearing, fire-purging, and heat-clearing, damp-drying principles, which are supplemented by drugs that benefit Qi and blood, nourish yin and engender fluids. To date, there have been extensive studies on the antiviral effects of TCM *in vitro*, which focus on single herbs such as *Lonicera japonica* Thunb. (Caprifoliaceae) flower (Jinyinhua), *Forsythia suspensa* Thunb. Vahl. (Oleaceae) fruit (Lianqiao), *Angelica dahurica* (Fisch. ex Hoffm.) Benth. et Hook. f. ex Franch. et Sav. (Umbelliferae) root (Baizhi), and *Curcuma zedoaria* (Christm.) Rosc. (Zingiberaceae) rhizome (Ezhu), as well as some of their active constituents (Law et al. 2017; Lee et al. 2020; Li, Xie, et al. 2020; Wan et al. 2020). Although their definite antiviral effects have been verified by *in vivo* or *in vitro* experiments, the specific mechanisms of their antiviral actions have scarcely been explored, where the emphasis is on the extraction of single herbal medicines. Flavonoids, polysaccharides, triterpenoids, alkaloids, etc. are mostly common constituents in the existing reports of antiviral TCM.

## Antiviral constituents in traditional Chinese medicines

Antiviral treatment with TCM has the advantages of broad spectrum, less adverse reactions, low drug resistance and holistic regulation. In recent years, the antiviral constituents in TCM and their mechanisms of action have been investigated extensively by scholars. Extant reports concerning the antiviral constituents of TCM centre on the alkaloids, flavonoids, polysaccharides, saponins, tannins and polyphenols. The research findings are summarized according to the structure type of constituents.

### Alkaloids in TCM

Alkaloids, as a class of nitrogenous organic compounds present in organisms, are usually alkaline in nature and have a broad range of activities. Regarding antiviral effects, they are resistant to multiple viruses including SARS-Cov, HCov-229E, EV71, influenza viruses, HIV, HBV, CVB3, etc., which are the main active constituents of high-frequency traditional Chinese medicines. *Isatis tinctoria* L. (Cruciferae) root (Banlangen) is widely used in TCM for curing diseases caused by bacteria and viruses such as influenza and other infections over thousands of years in China (Zhou and Zhang 2013; Xiao et al. 2016, 2017). Banlangen has been one of the eight major medicines which is recommended by the Chinese government for treating and preventing the deadly severe acute respiratory syndrome (Speranza et al. 2020). It played an important role in the prevention of severe acute respiratory syndrome in 2003 and swine flu pandemic in 2009 in China (Lin et al. 2005; Yang et al. 2012). Alkaloids were considered as one of the characteristic constituents of this drug, which possess diverse bioactivities such as antibacterial, antiviral, anti-inflammatory, antitumor, and antioxidant activities (Zhang, Shi, et al. 2019, Zhang et al. 2020a; Kang et al. 2020).

More than 100 alkaloids have been found in Banlangen by now, such as quinazolone alkaloids, indole alkaloids, quinoline alkaloids, and so on (Xi et al. 2019; Zhang et al. 2020b). Epigoitrin, a natural alkaloid from Banlangen, can prevent influenza virus infection by reducing the susceptibility of the host under stress. Researchers found that epigoitrin reduces susceptibility to H1N1 virus and proinflammatory cytokine production in stress-stressed mice to reduce pneumonia, and maintains MAVs antiviral signalling to ensure IFN-β production after H1N1 infection (Luo et al. 2019). Berberine is a natural isoquinoline alkaloids with extensive pharmacological activities including antidiarrhea, anticancer, antibacterial, anti-inflammatory and antiviral properties and was found in several medicinal plants, such as *Coptis chinensis* Franch. (Ranunculaceae) root (Huanglian), *Phellodendron amurense* Rupr. (Rutaceae) root (Huangbai) (Joshi et al. 2011; Samadi et al. 2020; Olleik et al. 2020; Yang et al. 2020). Berberine is a novel antiviral drug and therapeutic candidate for targeting different steps of viral life cycle and it can intercalate into DNA and inhibits DNA synthesis and reverse transcriptase activity. Berberine has the ability to inhibit the replication of herpes simplex virus (HSV), human cytomegalovirus (HCMV), human papillomavirus (HPV) and human immunodeficiency virus (HIV). This may occur through the inhibition of MEK/ERK signalling pathway, the activation of AMP-activated protein kinase (AMPK) and the inhibition of NK-κB (Warowicka et al. 2020). In addition, berberine supports the host's immune response, which leads to virus clearance (Han et al. 2020).

### Flavonoids in TCM

With complex, varied structural types, flavonoids are a series of compounds whose basic structure is 2-phenylchromone and they are divided into several classes such as flavonols, flavanones, isoflavones, flavones and anthocyanidins (Lalani and Poh 2020). They are widely used in TCM and have a variety of biological functions (Kumar and Pandey 2013). The pharmacological properties of flavonoids include anticancer, antioxidant, antibacterial, anti-inflammatory, and immunomodulatory functions (Bian et al. 2020). Flavonoids are the main active constituents of common antiviral traditional Chinese medicines such as *Scutellaria baicalensis* Georgi. (Labiatae) root (Huangqin), Jinyinhua and *Kaempferia galanga* Linn. (Zingiberaceae) rhizome (Shannai) and they have a wide range of antiviral effects on SARS-Cov, RSV, ADV, HCV, ZIKV, EBV, HSV-1, etc. (Shimizu et al. 2017; Chen, Zhang, et al. 2019; Jo et al. 2020; Wang, Cai, et al. 2020). Flavonoids have been studied against a variety of DNA and RNA viruses. They prevent the virus from entering the cell, interfere with various stages of viral replication or translation and multi-protein processing, and prevent the release of the virus from infecting other cells. Researchers found that different flavonoids inhibit the spread of the virus through different mechanisms. Table 1 summarizes the structure-activity relationship and mechanism of action of flavonoids against influenza virus.

**Table 1.** Components and mechanism of flavonoids in TCM against influenza virus.

Classification	Typical crude drugs	Efficacy	Main ingredients	Chemical construction	Mechanism
Flavone	*Scutellaria baicalensis* Georgi. (Labiatae) root (Huangqin)	Clearing away heat and promoting diuresis, cooling blood and clearing away toxic material	Baicalin	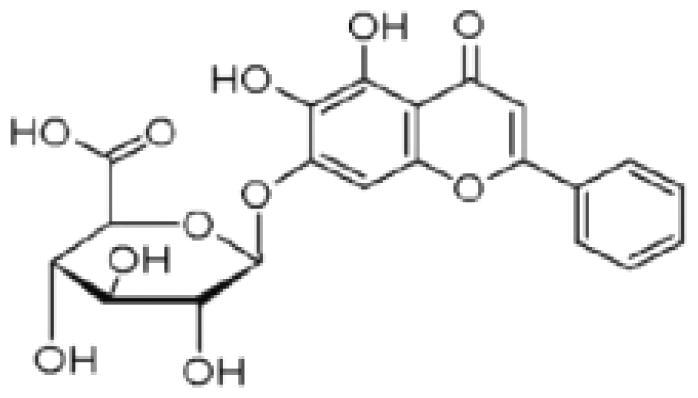	inhibit virus replication (Chu et al. 2015) inhibit NA activity (Ding et al. 2014) modulate viral protein NS1 (Nayak et al. 2014) reduce the pathological damage and inflammation of the lungs (Pang et al. 2018) regulate cell cycle and suppress the activation of caspase-8 and caspase-3 (Zhang et al. 2011) trigger macrophage M1 polarisation, IFN activation, and other cellular reactions (Geng et al. 2020) the inhibition of autophagy induced by virus (Zhu et al. 2015)
			Baicalein	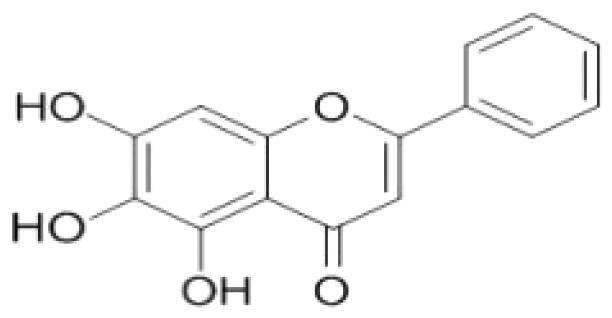	interfere NA activity (Sithisarn et al. 2013) inhibit the transcription and replication of mRNA of influenza virus (Hour et al. 2013) reduce expression of the viral matrix protein (Chen et al. 2011) reduce virus-induced cleavage of caspase 3 and reduced nuclear output of the viral RNP complex (Sithisarn et al. 2013) promote the formation of virus-induced intracellular reactive oxygen species (Michaelis et al. 2014)
			Wogonoside	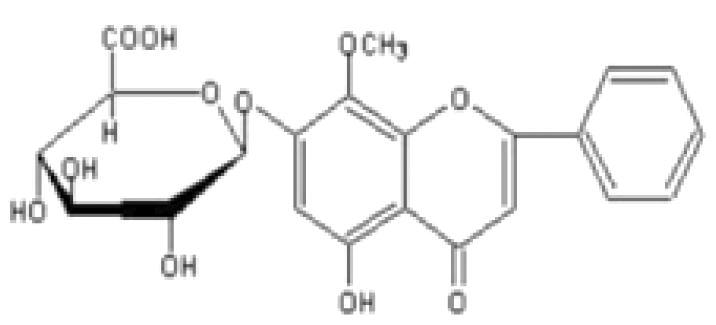	inhibit NA activity (Zhou et al. 2017)
			Wogonin	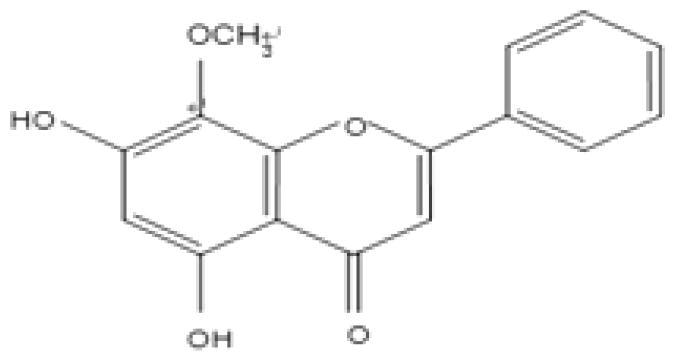	inhibit NA activity (Zhou et al. 2017) possesse a potent anti-influenza activity mediated by regulation of AMPK activation (Seong et al. 2018) inhibit the production of various inflammation - related factors in alveolar macrophages infected with influenza virus (Wu et al. 2011)
Flavonol	*Flos Sophorae* Immaturus Sophora japonica L. flower (Huaihua)	Clearing heat, cooling blood and stopping bleeding	Quercetin	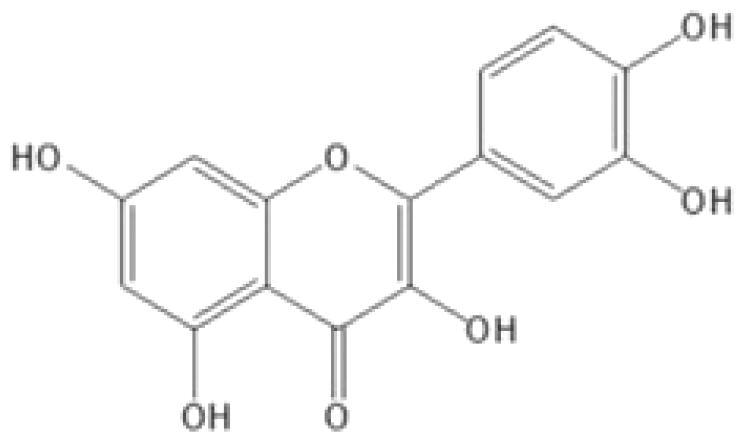	inhibit the entry of virus (Wu et al. 2015) protect the lung from the deleterious effect's of oxygen derived free radicals released during influenza virus infection (Kumar et al. 2005) inhibit the mRNA and protein expression of CDK4 induced by virus infection (Wan et al. 2013) anti-Inflammatory and immunomodulatory effects (Mehrbod et al. 2018) neuraminidase inhibitory activity (Lee IK et al. 2016) haemagglutinin inhibitory activity (Mehrbod et al. 2020)
			Kaempferol	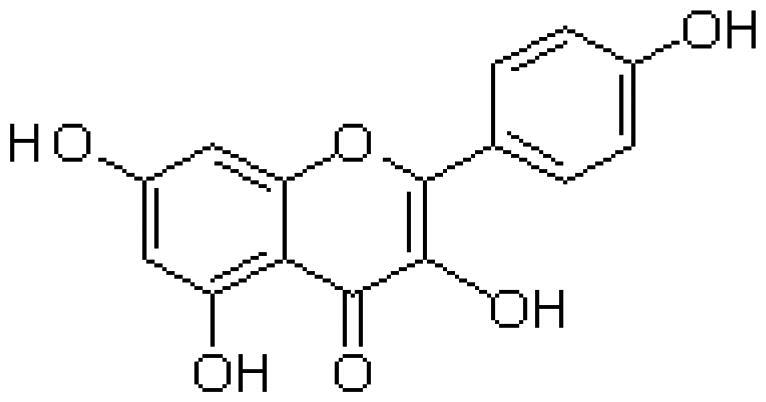	protective effect on virus-induced inflammation via suppression (Zhang et al. 2017) inhibit mRNA synthesis and viral protein expressions (Choi et al. 2019) suppress cell-autonomous immunity by down-regulate p38 and JNK expression and activation (Dong et al. 2014) promoted RNPs export by up-regulating ERK activation (Dong et al. 2014) inhibit NA activity (Rakers et al. 2014)
Flavonone	*Mentha haplocalyx* Briq. herb (Bohe)	Evacuating wind heat, clearing the leader, benefiting the pharynx and rash, soothing the liver and Qi.	Hesperidin	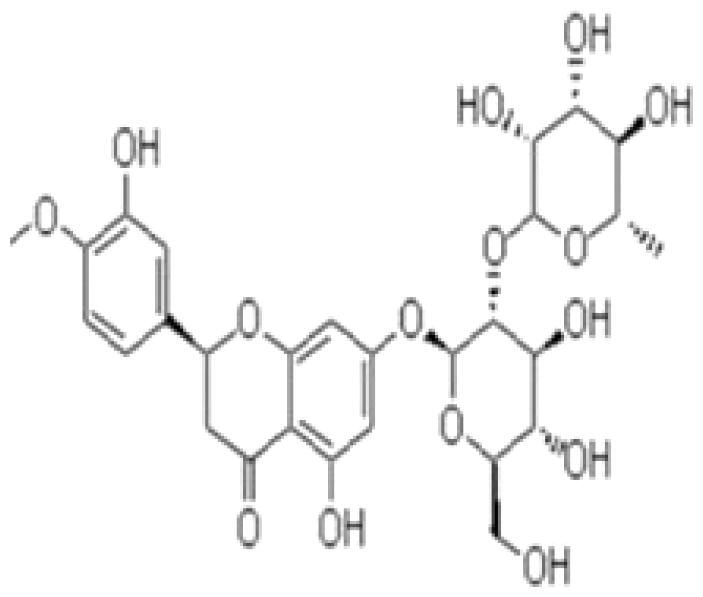	bind to the key entry or spike protein of virus (Montenegro-Landivar et al. 2021) alleviate H1N1-induced impairment of pulmonary function (Ding et al. 2018) enhanced cell-autonomous immunity (Dong et al. 2014) inhibit NA activity (Sharma et al. 2011) prevent replication by inhibition of viral sialidase activity that is involved in the entry and release stages on IAV infection (Saha et al. 2009)
Flavanonol	*Ampelopsis grossedentata* (Hand.-Mazz.) W. T. Wang tender leaf (Tengcha)	Clearing heat and dampness, calming liver and reducing blood pressure, promoting blood circulation	Dihydromyricetin	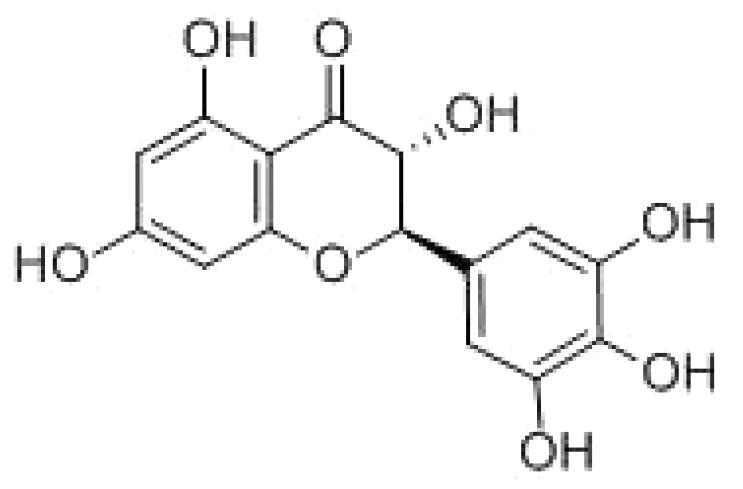	inhibit the replication of influenza A virus strains (Tian et al. 2020) reduced viral polymerase activity via selective inhibition of viral PB2 subunit, and decreased relative amounts of viral mRNA and genomic RNA during influenza A virus infection (Tian et al. 2020) inhibit absorption and uptake of cells (Roschek et al. 2009) reduce cellular immune injury by inhibiting TLR3 signalling pathway (Tian et al. 2020)
Isoflavones	*Astragalus membranaceus* (Fisch.) Bge. root (Huangqi)	Qi tonifying and superficial resistance strengthening, urination promoting to expel internal toxin/pus, tissue regeneration promoting and sore healing	Calycosin	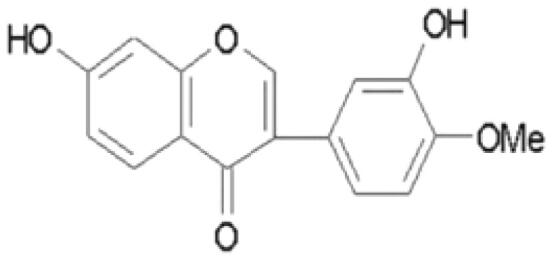	inhibit the increased permeability of the endothelial cells caused by influenza virus and the allosteric effect of F-actin, achieving the function of protecting endothelial cells (Zhang JJ et al. 2019)
Flavanol	Acacia catechu (L. f.) Willd. extract of branches (Ercha)	Promoting blood circulation and relieving pain, stopping bleeding and regenerating muscle, collecting dampness and heal sore, clearing lung and dissipating phlegm	Catechin	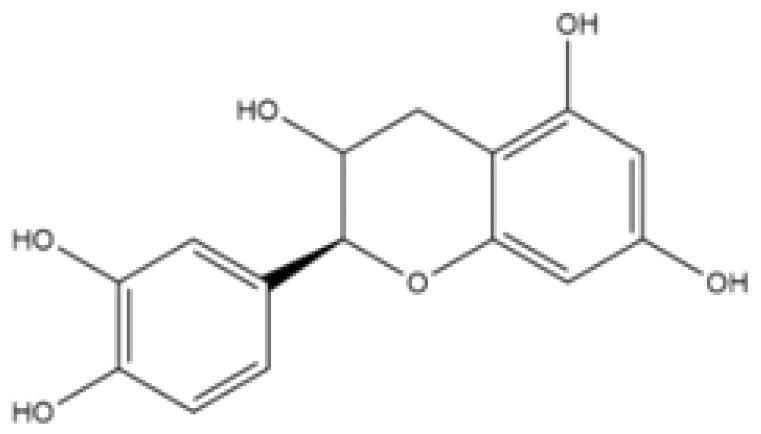	inhibit mRNA replication of influenza virus (Chang et al. 2020) inhibit NA activities (Muchtaridi et al. 2020) inhibit attachment of the host cell (Furushima et al. 2018) inhibit viral glycoprotein (You et al. 2018) inhibit autophagy induced by influenza virus (Choi et al. 2019)
			Epigallocatechin gallate (EGCG)	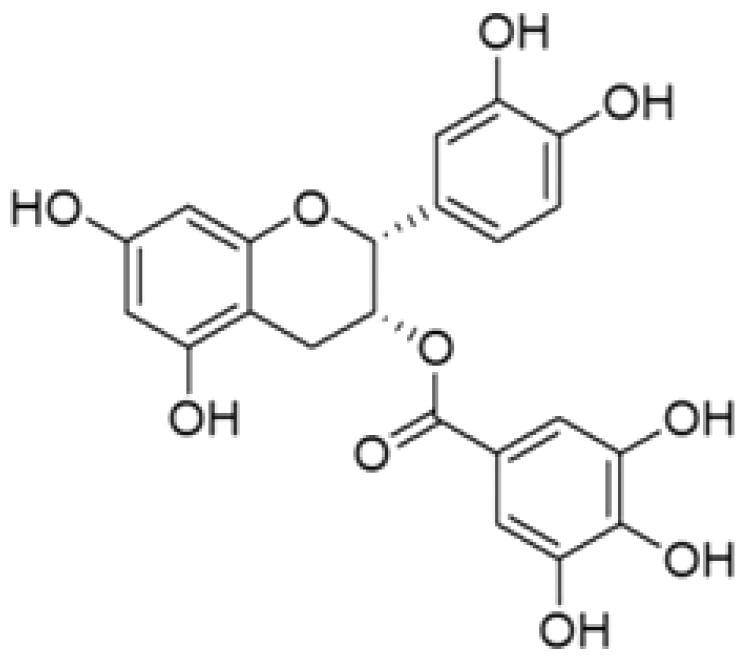	damage to the physical properties of the viral envelope (Kim et al. 2013) inhibite influenza A replication (Ling et al. 2012) remarkably downregulate TLR4 protein levels through 67LR/Tollip, decrease MPO activity and inflammatory cytokine levels (Xu et al. 2017) inhibit the early stage of infections, such as attachment, entry, and membrane fusion, by interfering with viral membrane proteins (Kaihatsu et al. 2018) inhibit NA activities (Ide et al. 2016)
			Epigallocatechin	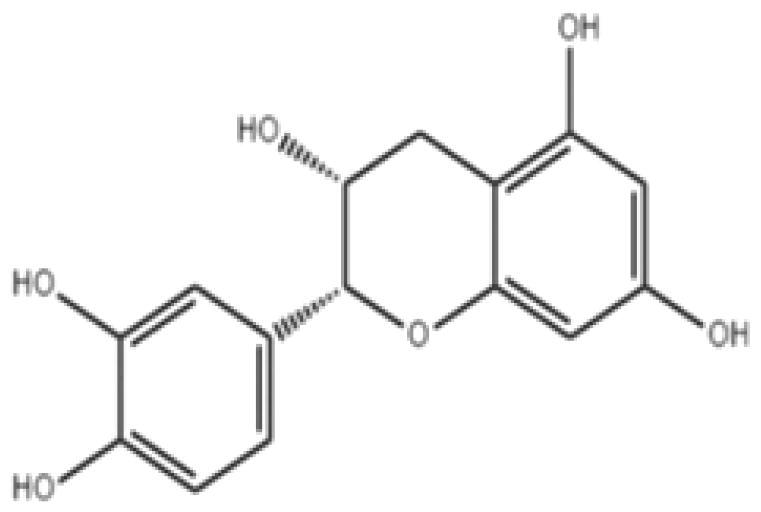	inhibit influenza A replication (Song et al. 2005) reduce virus entry into host cells (Imanishi et al. 2002) inhibit HA and NA activity of influenza virus (Song et al. 2005; Liu JX et al. 2012)

Based on antiviral action mechanisms, flavonoids can prevent inhibitors, treat inhibitors or indirect inhibitor systems that interact with the immune system. As one of the most popular TCM, Huangqin has a medicinal history at least 2000 years (Oo et al. 2019). Baicalin, baicalein, wogonin and wogonoside are the four major flavones in Huangqin and have various pharmacological functions, and have been widely investigated for their antiviral activities (Wang, Yang et al. 2020). Baicalein and baicalin down-regulated ZIKV replication 10 h after infection, and showed significant preventive effects in pre-treated cells. Baicalein showed the strongest effect on intracellular ZIKV replication, while baicalin showed the best inhibition effect on virus invasion (Guo et al. 2019). Modern studies have also found that baicalin can reduce viral titre in a dose-dependent manner *in vitro* without a direct virusicidal effect, it can also improve survival, reduce heart weight/weight ratio, prevent viral replication, and reduce myocardial inflammation in CVB3-induced acute viral myocarditis in a mouse model (Wang et al. 2018).

### Saponins in TCM

Saponins are a class of high molecular weight amphiphilic compounds having with sugars as hydrophilic moiety and triterpenoid or/and steroid aglycon as lipophilic moiety (Vincken et al. 2007; Singh and Chaudhuri 2018). They can be divided into triterpenoid saponins and steroidal saponins according aglycone structures. Saponins are widely existed in terrestrial higher plants, as well as in sea cucumbers, starfish and other marine organisms. Saponins are derived from oxidized polymers containing 30 carbon atoms, but the difference is that the triterpenoid saponins have 30 carbon atoms retained, while the steroid saponins have 3 methyl groups removed (He et al. 2019).

Saponins have a wide range of pharmacological effects and are considered to be the effective components of many traditional Chinese medicines, such as *Panax pseudo-ginseng* Wall. var. *notoginseng* (Burkill) Hoo & Tseng (Araliaceae) root (Sanqi), *Anemarrhena asphodeloides* Bunge (Liliaceae) rhizome (Zhimu), *Bupleurum chinensis* DC (Umbelliferae) root (Chaihu), etc. (Nian et al. 2017; Zhou et al. 2019; Feng et al. 2020). The saponins in many traditional Chinese medicine, such as *Panax ginseng* C. A. Meye*r* (Araliaceae) root (Renshen), *Glycyrrhiza uralensis* Fisch. (Leguminosae) root (Gancao), *Astragalus membranaeus* (Fisch.) Bge. (Leguminosae) root (Huangqi) etc. have good antiviral effect (Ganesan et al. 2015; Gado et al. 2019; Alexyuk et al. 2019). Renshen has been frequently used as traditional medicine and healthy food in Asia with a long history and it contains a large amount and number of ginsenosides (Zhang, Abid, et al. 2020). There are more than 123 saponins reported in Renshen from 11 different species including both naturally occurring compounds and those from steaming and biotransformation (Yang et al. 2014; Piao et al. 2020). Modern research results show that ginsenosides have the potential to against CVB3, EV71, HRV 3 C, HSV, haemagglutinating virus of (HVJ) infection, and influenza A (Molnar et al. 2000; Song et al. 2014; Dong et al. 2017). Ginsenoside 20(*S*)-Rg3 inhibited HSV-1 and HSV-2, and the IC_50_ was about 35 μM. Inhibition was strongest when the virus was exposed to 20(*S*)-Rg3 for 4 h prior to cell addition. Saponins are promising as a potential chemotherapeutic agent for the treatment of herpes simplex virus, and when used in combination with valacyclovir may prevent an increase in drug resistance and affect some aspects of the interaction between the virus and host receptor proteins (Wright and Altman 2020).

### Volatile oils in TCM

Volatile oils, with monoterpenoids and sesquiterpenoids as the major components, refer to a class of aromatic oily liquids, which are volatile at room temperature and can be distilled with water vapour (Edris 2007). Volatile oils possess a broad spectrum of physiological activities, which are the main active constituents of many common antiviral traditional Chinese medicines such as *Nepeta cataria* Linn. (Labiatae) whole herb (Jingjie), *Houttuynia cordata* Thunb. (Saururaceae) overgroud part (Yuxingcao) and Lianqiao (Yuan et al. 2017; Lee, Wang et al. 2018; Řebíčková et al. 2020). A large number of studies have shown that volatile oils have antioxidant, antibacterial, antifungal and antiviral effects (Zhang, Huo, et al. 2020; Darwish et al. 2020; El-Alam et al. 2020). Because of the lipophile nature of the volatile oils, they are advocated to penetrate the viral membrane easily leading to membrane rupture. Volatiles oils contain a variety of active chemical components that synergize multiple stages of viral replication and have positive effects on the host respiratory system, including bronchiectasis and mucolysis (Youssef et al. 2020).

The volatile oil of TCM is active to many kinds of viruses, especially enveloped viruses, such as herpes simplex virus, HIV virus, IFV virus and avian influenza virus (Ma and Yao 2020). Ginger has been used in Asia for centuries to ward off colds and colds because of its antiseptic and anti-inflammatory properties. In Europe, ginger is used for its digestive properties and its therapeutic effect on the digestive tract (Camero et al. 2019). The inhibitory effect of essential oil of ginger on RC-37 cells of herpes simplex virus type 2 (HSV-2) *in vitro* was screened by thrombocytopenia. The results showed that the effect of volatile oil on HSV-2 mainly occurred before the adsorption, possibly through the interaction with the viral envelope (Koch et al. 2008). The researchers tested the efficacy of peppermint essential oil derived from *Mentha haplocalyx* Briq. (Labiatae) aerial portion (Bohe) (EOMS) and its active principle piperitenone oxide (PEO) *in vitro* experimental model of infection with HSV-1. The results showed that the antiviral activity of the two compounds was mainly due to the effect of viral adsorption. Among them, PEO exerted a strong inhibitory effect by interfering with the late stage of HSV-1 life cycle. The compound may interfere with some REDOX sensitive cellular pathways involved in viral replication (Civitelli et al. 2014). These results suggest that EOs may be a potential therapeutic agent for the treatment of viral infections, as well as a prototype for new antiviral drug options, such as their proposed activity against SARS-CoV-2 virus (Asif et al. 2020).

### Polysaccharides in TCM

Composed of carbon, hydrogen and oxygen, polysaccharides are a class of important biological macromolecular substances that are ubiquitously present in the cells of animals, plants and microorganisms. In recent years, polysaccharides have attracted more and more attention because of their wide biological activity and high safety (Shi 2016; Muhama et al. 2019). Scientific advances have proven that polysaccharides have various biological activities including antioxidant activity, up-regulation of immunity, anticancer activity antiviral activity and etc., with less adverse effects (Chen, Zhang, et al. 2020; Gu et al. 2020; Dong, Hou et al. 2020; Li, Xiang, et al. 2020). Several natural polysaccharides, such as spruce polysaccharides and fucoalgae polysaccharides, have been used in clinical health care and treatment of diseases (Wang et al. 2019; Chen, Li, et al. 2020). Many polysaccharides from different sources possess antiviral activities, which are the main active constituents of common antiviral traditional Chinese medicines such as *Portulaca oleracea* Linn. (Portulacaceae) whole herb (Machixian), *Stevia rebaudiana* L, and Huangqi (Liu, Niu, et al. 2020). *Prunella vulgaris* Linn. (Labiatae) spike (Xiakucao) is well known as a traditional Chinese drug for its effects on treating oral ulcer, hypertension and also as an astringent for external and internal purposes (Fang et al. 2019). Modern reports have shown that polysaccharide in Xiakucao has antiviral effect, especially good curative effect on HSV (Ma et al. 2016). Research shows that the purified polysaccharide of Xiakucao is composed of galactose and sulphate-containing glucose, and they are an HSV inhibitor. They can greatly reduce the number of infected viruses, hinder the absorption of viruses, and can directly inactivate the virus in the cell (Xu et al. 1999; Zhang et al. 2007). In addition, researchers also found the polysaccharide of Xiakucao reduced the repression viral antigen against acyclovir sensitive virus strain (Chiu et al. 2004). The above findings indicate that polysaccharides of traditional Chinese medicines have great potential in antiviral research.

### Tannins and other phenols in TCM

Tannins are a class of water-soluble polyphenolic compounds with protein precipitation properties, which have antiviral effects apart from common activities such as astringent, antibacterial, anti-inflammatory, haemostatic, antidiarrheal, as well as anti-infective against multiple pathogens (Ogawa and Yazaki 2018; Brossard et al. 2020; Ma et al. 2020). Polyphenols, on the other hand, refer to the plant constituents with several phenolic hydroxyl groups in their molecular structure, which are known as the ‘seventh nutrients’. Hydrolysed tannins, condensed tannins and chloroplast tannins are the main tannins in different parts of higher plants (Watrelot and Norton 2020). They possess certain antiviral effects, such as curcumin and bisdemethoxycurcumin (both with anti-influenza effects) in *Curcuma longa* Linn. (Zingiberaceae) root (Jianghuang) and resveratrol in *Paeonia lactiflora* Pall (Paeoniaceae) root (Baishao). Extensive research indicates that Baishao has a variety of pharmacological actions, especially its effects on the blood vessels and heart and liver protection (Chen et al. 2015; Xie et al. 2018; Tan et al. 2020). Baishao was used for Wen Bing (Warm Disease) treatment in ancient China. Three tannins in the aqueous extract of *Paeonia lactiflora* were found to inhibit the replication of influenza A virus in MDCK cells, and all three compounds significantly reduced the activity of neuraminidase (Zhang, Lo, et al. 2020). Recent studies have shown that porcine jujube exhibits antiviral activity against DENV-2 in Vero cells, and its anti-dengue properties are most likely attributable to its tannin compounds (Pong et al. 2020).

### Other antiviral constituents in TCM

Apart from the aforementioned constituents, phenylpropanoids, anthraquinones and organic acids have also been reported to possess antiviral activities. In addition to alkaloids, Banlangen also contains a variety of other antiviral chemical components. Chemical studies showed that it contains various phenylpropanoid, such as clemastanin B, indigoticoside A and syringin are the most abundant compound isolated from Banlangen (Peng et al. 2005; Xiao et al. 2014). Recent studies have shown that clemastanin B has different inhibitory activities against different subtypes of human influenza viruses and avian influenza viruses (Yang et al. 2013). Researchers also found that clemastanin B has anti-influenza activity by therapeutic, cell-preventive and inhibits viral attachment, but does not have any direct virulent activity (Xiao et al. 2016). Some examples are emodin in *Rheum officinale* Baill. (Polygonaceae) root and rhizome (Dahuang) and organic acids in Jinyinhua, all of which are from common antiviral traditional Chinese medicines (Ding, Cao, et al. 2017; Ren et al. 2019; Wu et al. 2019; Dong, Zeng et al. 2020).

## Antiviral mechanisms of traditional Chinese medicines

Antiviral treatment with TCM can be achieved in two ways, namely, the direct antiviral therapy (eliminating pathogens) and the indirect antiviral therapy (strengthening vital energy). With pathogen elimination, the drugs inhibit or disrupt the viruses directly. In the meanwhile, strengthening of vital energy exerts an antiviral role by regulating and enhancing the body immunity from a holistic perspective, which is a non-negligible aspect of the antiviral mechanisms played by TCM. A combination of pathogen elimination and vital energy strengthening can take the individual differences into account and avoid the viral mutation problem, thereby allowing better antiviral efficacy of TCM.

**Figure 1.**
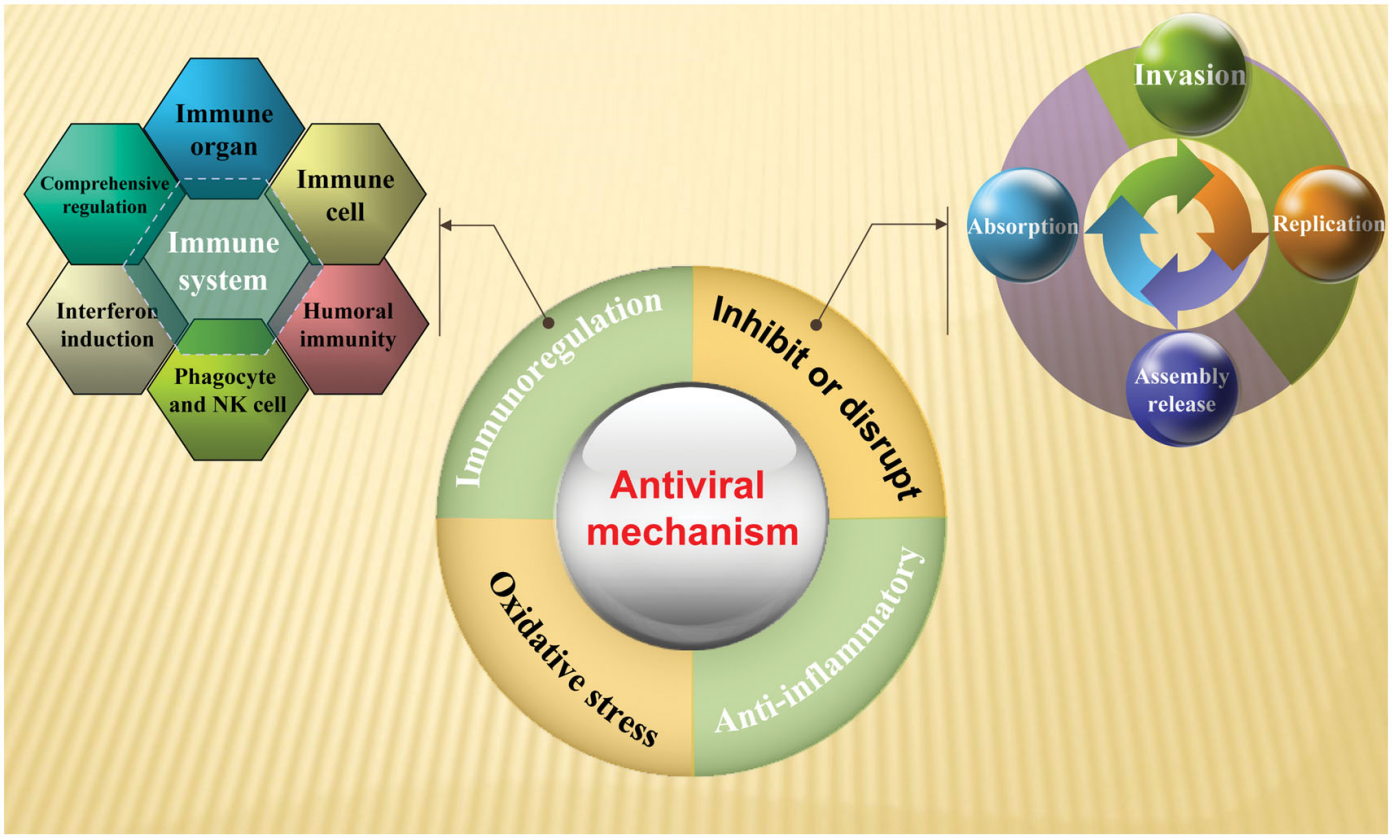
Antiviral mechanism of traditional Chinese medicine.

In recent years, people’s understanding of viral replication and pathological mechanisms has deepened gradually, which has laid a foundation for further R&D of antiviral traditional Chinese medicines. Viral proliferation process consists of adsorption to host cells, invasion, uncoating, genome replication, protein synthesis, assembly, and release, etc. Antiviral mechanisms of Chinese herbal medicines achieve inhibition of viral proliferation primarily by intervening at one or more stages of viral replication inside the host cells. Besides, they can also promote the development of immune organs, enhance the activity of immune cells, and induce the secretion of numerous antiviral proteins. According to modern research findings, a multitude of Chinese herbal medicines are capable of inducing interferon (IFN) and interleukin-12 (IL-2) production in organisms, two cytokines that are crucial to the antiviral immunity. When IFN binds to the IFN receptor on the cell membrane, the genes encoding antiviral proteins are activated, followed by synthesis of antiviral proteins. Besides, Th1 cellular immunity can also be induced to exert non-specific antiviral effects. Additionally, the antiviral proteins can also be developed as antiviral immunopotentiators. Despite multiple pathways to fight viruses with traditional Chinese medicines, the key to controlling viral infections lies in the inhibition of viral replication by exerting the body's conditioning mechanisms. The common mechanism of TCM is shown in Figure 1.

### Inhibition of viral adsorption and invasion

Cell membrane is the first barrier before the cellular invasion by viruses. Adsorption and invasion refer to the viral attachment onto the surface of susceptible cells, and the subsequent penetration of cell membrane by viral nucleic acids or infectious nucleocapsids to enter the cytoplasm. This is the initial stage of infection. Flavonoids, triterpenoids, polysaccharides and their derivatives in traditional Chinese medicines are all effective in inhibiting the adsorption of viruses. The glycyrrhizin (GL) and its aglycone 18 β-glycyrrhetinic acid are the most thoroughly studied active substances of Gancao (Li, Liu, et al. 2020). They are reported to have antitumor, antioxidant, anti-inflammatory and antiviral properties (Cai et al. 2019; Shafik et al. 2019; Bailly and Vergoten 2020; Shiba et al. 2021). The mechanism of GL protecting cells from influenza A virus (IAV) infection was studied, and the antiviral effect of GL on a variety of IAV infected cells was demonstrated. The antiviral effect of GL was found to be limited to the early stages of the viral replication cycle. The direct inhibition of GL on IAV particles was excluded without binding to the virus receptor, suggesting that GL inhibition of viral uptake is most likely to eventually lead to a reduction in engraftment by interfering with the cell membrane. Therefore, GL can be referred to as a virus inhibitor rather than a virus killer (Aguilar et al. 1999).

Polysaccharides are similar to cell receptor heparin sulphate, which is necessary for viruses to absorb and infiltrate cells. In the presence of polysaccharides, viruses seem to bind to these molecules rather than to cell receptors. Researchers found polysaccharides can compete with virus adsorption and osmosis (Cheshenko and Herol 2002; Wolkerstorfer et al. 2009; Ekblad et al. 2010). *Stevia rebaudiana* (Bertoni) Hemsl (Asteraceae) is a perennial herb (Zhang, Liu, et al. 2020). Modern studies have shown that it has antioxidant, antiamnesic, antidiabetic, thrombolytic, cytotoxic and antibiofilm activities that might be significant for the management and treatment of various diseases (Noreen et al. 2020). The mechanism of two polysaccharides with anti-HSV-1 activity was studied using stevia leaf as raw material. Both exert antiviral effects at the initial stage of HSV-1 infection by inhibiting viral adsorption and osmosis, and this activity is directly related to the interaction between stevia polysaccharides and viral glycoproteins, rather than cell receptors. Polysaccharides extracted from *S. rebaudiana* may be an alternative treatment for HSV-1 infection. Even if this treatment does not eliminate latent HSV-1, it can prevent transmission of the virus when the first symptoms appear (Ceole et al. 2020).

### Inhibition of viral replication

Nucleic acids are the genetic material of viruses. Depending on the type of genetic material, viruses can be classified into two categories: DNA viruses and RNA viruses. For antiviral drugs, prevention of viral nucleic acid replication is an effective target.

Polymerase and helicase protein are major targets for DNA and RNA synthesis and replication inhibitors. RNA-dependent RNA polymerase (RdRp) is an indispensable enzyme in the genome replication and transcription cycle of RNA viruses (Shadrick et al. 2013; Venkataraman et al. 2018). Helicase is a motor protein that separates and rearranges nucleic acid duplexes in processes powered by adenosine triphosphate (ATP) hydrolysis. The helicase protein unwinds double-stranded DNA and RNA into single strands so that they can be copied (Ivanov et al. 2004).

Currently, ion channel blockers, polymerase inhibitors and helicase inhibitors are the common western drugs for suppressing viral nucleic acid replication. TCM has certain advantages in inhibiting viral replication and reducing viral load. Most of such prescriptions are pathogen dispelling drugs, as well as alkaloids, flavonoids, etc.

*Tussilago farfara* Linn. (Compositae) flower (Kuandonghua) is a perennial herb with thin branching rhizomes and yellow flowers (heads) before flowering in early spring (Liu, Wu, et al. 2020). It is recorded in all versions of the Chinese pharmacopoeia and is used in many proprietary Chinese medicines to treat cough, sputum, bronchitis and asthma (Li et al. 2012; Xue et al. 2012; Shikov et al. 2014). It has several other biological activities such as effects on the digestive and cardiovascular systems, anti-inflammatory, antioxidant, neuroprotective activities and antiviral activity (Kim et al. 2006; Lee et al. 2010; Lee, Kang, et al. 2016; Lee, Song et al. 2018). The study shown that Kuandonghua could effectively inhibit EV71-induced cell injuries by preventing protein expression and viral replication (Chiang et al. 2017). *Dryopteris crassirhizoma* Nakai (Dryopteridaceae) root and rhizome (Mianma Guanzhong) and *Morus alba* L. (Moraceae) leaf (Sangye) contain different flavonoids, some of which have been reported to have anti-dengue potential *in vitro*. The experiment discovered two kinds of plant extracts as the forerunner of dengue extract, for all DENV serotype has inhibitory effect. The extracts of *Dryopteris crassirhizoma* were most effective in the late stage of virus replication, while the extracts of *Morus alba* were most effective in the early stage of virus replication and higher doses might even have prophylactic activity. This led to the idea of using TCM to treat the different stages of viral replication (Maryam et al. 2020). Baizhi is used as a traditional folk medicine in China for headaches, abscesses, fever, the common cold, flu, etc. (Sarker and Naharl 2004; Li et al. 2021). In recent study, researchers isolated four representative anti-influenza activities of furanocoumarins from 70% ethanol extract of *Baizhi*. The four isolates showed dose-dependent CPE inhibitory activity against H1N1 and H9N2. Compound 2 interferes with the synthesis of NA and NP at the early stage of the viral replication cycle, but does not affect the entry of the virus into host cells, the emergence and release of the virus, nor the virulent activity of the virus, showing anti-influenza activity (Lee et al. 2020).

Researchers studied the anti-SARS activity of *Houttuynia cordata* Thunb (Saururaceae) aboveground part (Yuxingcao) and detected its effect on RdRp activity by using filter combined with polymerase chain (Lau et al. 2008). The results showed that Yuxingcao exhibited significant inhibitory effects on SARS-CoV 3 C-like protease (3CLpro) and RNA-dependent RNA polymerase (RdRp), suggesting that Yuxingcao had an inactivation effect on the RdRp activity of SARS coronavirus (Lau et al. 2008). A recent study found the inhibitory effects of three main chemical substances curcumol, curdione and germacrone on influenza virus. *In vivo*, these compounds reduced H1N1-induced lung damage and viral load in serum as well as the whole blood cells, germacrone was also found to inhibit both the viral attachment/entry step and the early stages of the viral replication cycle (Liao et al. 2013; Li, Xie, et al. 2020). In proteomic analysis, the expression of antiviral protein and intracellular virus were significantly reduced after germacrone treatment, further proving that gemacrone can inhibit viral replication (Li, Xie, et al. 2020).

### Inhibition of viral assembly and release

Assembly and release of viruses constitute the last step of viral replication, where the mature virus particles are released from host cells in the form of exocytosis and budding. So far, there have been few reports of TCM acting on this stage. Virus envelope (E), membrane (M), nucleocapsid (N), and some accessory proteins play an important role in the assembly of virions. Therefore, preparations targeting their specific binding sites or functions have broad-spectrum activity against viruses (McBride and Fielding 2012; Klingler et al. 2020). COVID-19 caused by the SARS-CoV-2 was discovered in December 2019. Since its emergence, the virus has exploded around the world, killing hundreds of thousands of people and infecting a growing number in what the World Health Organization (WHO) has called a pandemic (Wu et al. 2021). At present, there is an urgent need to develop a suitable specific drug against New Coronavirus. Encouragingly, more and more TCM has been found to target specific binding sites of the proteins in the assembly of virions (Qiu et al. 2020). For example, the accessory open-reading-frame 3a protein has previously been shown to form a cationic selective channel that may be expressed in infected cells and participate in the release of the SARS-Cov (Yue et al. 2018). Therefore, drugs targeting the channels formed by accessory protein 3a are expected to inhibit viral release (Zhang, Yu, et al. 2020). Emodin is an anthraquinone compound extracted from Dahuang and has been demonstrated to possess anti-inflammatory, antioxidant, immunosuppressive, antitumor and antiviral activities (Ahn et al. 2015; Dai et al. 2017). Modern research finds that emodin is an ion channel inhibitor of SNE-encoded accessory 3a protein (Schwarz et al. 2011). In addition, studies also have shown that emodin not only destroys the viral envelope during viral release, but also leads to a decrease in RNA, suggesting that emodin is dependent on inhibiting viral release (Ho et al. 2007).

### Immunomodulators

Pathogeneses of many viral diseases are dominated by the pathological impairment of immune response resulting from the direct viral invasion and damaged tissues (Yang et al. 2021). Immune response is classified into specific and non-specific. The physiological significance of immune response to organisms is threefold: defense, homeostasis, and surveillance (Herich et al. 2019). Defense means to prevent viruses from invading and to clear them away timely. Homeostasis means sustaining the dynamic equilibrium of immunity, where the damaged or necrotic cells resulting from virus infection are eliminated by immunity. As for surveillance, it aims to detect diseased cells in the body at any time and to remove them timely. Coordination and effectiveness of these three functions are reliant on precise immunoregulation, while viral infection often leads to immune dysfunction. TCM is composed of many ingredients and often plays a role through various mechanisms, which not only targets at the virus, but also the host's immune response and produces a synergistic effect (Ding, Zeng, et al. 2017; Zhang, Morris-Natschke, et al. 2020).

In thousands of years of clinical practice, Banlangen have been widely used to treat seasonal influenza. The extracts of Banlangen have significant inhibitory effects on various subtypes of avian influenza virus and also inhibit degradation of Iκ-Bα and production of PGE2, NO, and interleukin 16 in LPS-stimulated RAW264.7, indicating that Banlangen plays an immune regulatory role *in vitro* and *in vivo* (Shin et al. 2010; Yang et al. 2012). *Commelina communis* Linn. (Commelinaceae) whole herb (Yazhicao) has been used as an antipyretic or diuretic in TCM, and is often used for common cold, high fever, sore throat, sore throat, edoema and oliguria, sore pain in hot bath, boils and swelling (Bing et al. 2009; Zhang et al. 2018). Homonojirimycin from Yazhicao exhibited antiviral activity against H1N1 compared with ribavirin (Zhang et al. 2013b). In addition, modern studies have identified the effect of homonojirimycin on influenza virus infection in mice, showing that it prolongs the average survival time, improves survival, and reduces the production of the virus in the lungs. In the study of biological mechanisms, homonojirimycin have protective effects against influenza virus infection and generate an effective immune response in the body, which may help to prevent or treat influenza virus infection (Zhang et al. 2013a).

Ge Gen Decoction (GGD) is widely used in clinical practice in Asian countries, and no adverse reactions have been reported so far. Experimental studies and clinical practices have found that it can relieve influenza-like symptoms (Kurokawa et al. 2002). Researchers demonstrated that GGD has anti-IAV H1N1 activity *in vivo* and *in vitro*. GGD down-regulates the expression of TNF-pro-inflammatory cytokines, regulates Th1/Th2 immune balance, reduces inflammatory response, and improves the prognosis of H1N1 infected mice. TLR7 signalling pathway is also involved in the immune regulation of GGD therapy. It is suggested that GGD has unique anti-inflammatory and immunomodulatory effects, and may be an effective anti-IAV drug (Geng et al. 2019).

### Antiviral therapy targeting host cells

Viruses regulate the host cell cycle through a series of interactions with host cells by using various self-encoded multifunctional proteins, thereby creating favourable conditions for replication to fulfil multiple functions. Viral infections and their induction of cytokines can trigger pro-apoptotic stimuli to induce apoptosis to limit dissemination, viral replication, and/or persistent infection of cells (Shore et al. 2011). EV71 belongs to the enterovirus family and is an uncoated, small single-stranded, positive-sense RNA virus (Yen et al. 2018). EV71 is a seasonal pathogen of gastrointestinal tract, causing secondary viremia, and it has become an important aetiology of central nervous system infections (McMinn 2002; Solomon et al. 2010). Infection of EV71 can manifest aseptic meningitis, flaccid paralysis, rhombencephalitis with myoclonus and ataxia or tremor, and death (Wang et al. 2017; Good et al. 2019). After infection with EV71, the level of Bax protein increased and apoptosis was induced (Kuo et al. 2002; Zhang et al. 2014; Li et al. 2015). Gan-Lu-Siao-Du-Yin (GLSDY) is a famous prescription of TCM with a history of more than 1000 years; it is effective in treating manage endemic diseases such as fever, fatigue and sore throat with ulcers. In recent years, GLSDY has been empirically used to manage enteroviral infection (Yen et al. 2018). Researchers used human foreskin fibroblast cell (CCFS-1/KMC) and human rhabdomyosarcoma cell (RD cells) to demonstrate that GLSDY can effectively inhibit EV71 infection by directly inhibiting caspase-8 and indirectly inhibiting Bax protein (Hsieh et al. 2016).

### Anti-inflammatory effect

Inflammation aggravates the immune inflammatory injury of virus-infected target organs. Inflammatory response remains one of the most common and serious complications of disease (Xie et al. 2019). A growing body of evidence suggests that in addition to direct viral damage, uncontrolled inflammation caused by host immune response disorders can also lead to disease severity and death (Caiazzo et al. 2020; Moore and June 2020; Cao and Li 2020). Under physiological conditions, anti-inflammatory cytokines regulate and balance the inflammatory response. However, the dual function of cytokines can be beneficial or harmful to the host. Under pathological conditions, the proinflammatory response is out of control and the balance is disrupted, and excessive proinflammatory cytokines and inflammatory immune cells may lead to additional tissue damage and inflammation (de Jong et al. 2006).

Agstragaloside IV, a major active compound extracted from the root of Huangqi, has extensive pharmacological activities, including anti-inflammatory, antioxidative, antiviral and anti-cancer functions (Liu, Shang, et al. 2020; Ge and He 2020; Hu et al. 2021). Studies shown that agstragaloside IV stimulates the formation of autophagosomes and its fusion to lysosomes in H1N1 infection. Although agstragaloside IV did not affect viral replication, it significantly reduced the secretion of IL-1β, possibly enhancing autophagic flux (Zhang, Zhang, et al. 2020). Lianhuaqingwen, a Chinese paten medicine composed of 13 herbs, has been used frequently in treating pneumonia, early stage of measles and bronchitis, and it played a positive role in the treatment of SATS-CoV-2 (Fang et al. 2020; Khan et al. 2020). New research suggests that Lianhuaqingwen significantly inhibited the replication of SARS-CoV-2 in Vero E 6 cells and significantly reduced pro-inflammatory cytokines (IL-6, TNF-α, CCL-2/MCP-1 and CXCL-10/IP-10) at the mRNA levels. These findings indicate that Lianhuaqingwen resist the attack of the virus, which indicates that this is a novel strategy for controlling the COVID-19 disease (Li RF et al. 2020).

### Other mechanisms

There is a close association of viral infection with oxidative stress. Organisms maintain the internal homeostasis by keeping the balance between oxidants and antioxidants, while viral infections cause an imbalance of oxidants and antioxidants in organisms, thereby resulting in oxidative damage to tissues and cells. Research has shown that icarisid can induce elevated expressions of GSH, GST and SOD1 in HepG2.2.15 cells *in vitro*, which also exerts an antioxidant action via antioxidant enzymes and non-enzymatic antioxidants that act on antioxidant system. Presumably, its anti-HBV effect is linked to the oxidative stress resistance (Xiao 2015).

## Summary and future prospects

TCM has become the focus of antiviral research because of its advantages of reliable clinical efficacy, fewer side effects and low drug resistance, which are derived from the holistic concept and the principle of syndrome differentiation. Holistic treatment of TCM focuses on the interaction and relationship among the body, virus, and drugs. It has considerable adaptability and effectiveness advantages in the treatment of complex human diseases that cause immune imbalance, especially in the case of the outbreak of unknown new viruses. This holistic approach is also being applied to emerging fields of network pharmacology, such as network biology and metabolomics. Another characteristic of TCM is the combination of multiple components and drugs, which often has multiple effects. The antiviral mechanism of TCM is not single, but the coexistence and interaction of many mechanisms. For example, Lianhua Qingwen (LQC) has been widely recommended throughout China for the treatment of COVID19 (Zheng, Zhang, et al. 2020). Research has found that the main functions of LQC include inhibition of virus binding to host cells, inhibition of cell replication and release, reduction of chemokine/cytokine expression levels, enhancement of immune system, and improvement of symptoms caused by different diseases (Shen and Yin 2021). Great progress has been made in antiviral research of TCM, but there are still many problems to be solved.

1. The lack of research on chemical composition analysis and antiviral mechanism of Chinese medicine restricts its development and is also an obvious shortcoming of modern TCM system. The chemical composition of TCM is complex, the specific mechanism of action and target of action are not completely clear, and the dose–effect relationship is not completely determined. Different delivery routes of the same drug may have different antiviral immune mechanisms, all of which require more in-depth experimental studies. Viral infection often leads to apoptosis. The present study showed that the protection of the host cell is one of the important ways to play antivirus activity of TCM. However, the moderate apoptosis caused by virus infection is actually a way for the host to prevent virus replication or diffusion in the infected cells, and also a means for the body to prevent virus diffusion. The antiviral effect of TCM on the prevention of apoptosis should be further studied.
2. The further development of TCM in the world must clarify its toxic and side effects. Research on the safety of TCM treatment of viral diseases is not enough. The ‘safety’ of TCM is not the same as ‘natural’. Some Chinese medicines have endogenous toxicity to liver, kidney and other organs, and exogenous toxicity is also introduced in cultivation, processing, storage and distribution. In addition, the complex chemical composition determines that some adverse reactions may occur in the process of treating diseases, especially in TCM injections. Adverse reactions may be caused by the active ingredients themselves or their decomposition products, impurities not removed in the preparation process, and toxic ingredients produced by the interaction of various components after combined use with other antiviral drugs.
3. Combination of Chinese and Western therapy. The antiviral mechanism of TCM is summarised and analysed, and it is found that the multi-components and multi-targets of TCM interact with each other. In the future study, on the premise of thoroughly studying the material basis of TCM effect, specific targets of TCM action against pathogens or the body should be identified. By exploring the structure–activity relationship and the overall effect of each active ingredient, and taking advantage of the characteristics of multiple components and multiple targets of TCM, the combined use of multiple components of a single drug or the combined application of Chinese and Western antiviral drugs were developed. At the same time, using the methods of evidence-based and pharmacological analysis of Western medicine for reference, we can accelerate the screening of safe and effective TCM through scientific data analysis.
